# Cranio-Facial Characteristics in Children with Autism Spectrum Disorders (ASD)

**DOI:** 10.3390/jcm8050641

**Published:** 2019-05-09

**Authors:** Gabriele Tripi, Sylvie Roux, Domenica Matranga, Laura Maniscalco, Pasqualino Glorioso, Frédérique Bonnet-Brilhault, Michele Roccella

**Affiliations:** 1 Department Promozione della Salute, Materno-Infantile, di Medicina Interna e Specialistica di Eccellenza “G. D’Alessandro”-PROMISE-University of Palermo, 90127 Palermo, Italy; domenica.matranga@unipa.it; 2 Childhood Psychiatric Service for Neurodevelopmentals Disorders, Centre Hospitalier du Chinonais, 37500 Saint-Benoît-la-Forêt, France; p.glorioso@ch-chinon.fr; 3 UMR 1253, iBrain, Université de Tours, Inserm, 37000 Tours, France; sylvie.roux@univ-tours.fr (S.R.); frederique.brilhault@univ-tours.fr (F.B.-B.); 4 Department of Biomedicine, Neuroscience and Advanced Diagnostics-BIND-University of Palermo, 90127 Palermo, Italy; laura.maniscalco04@unipa.it; 5 Department of Psychological Sciences, Pedagogical and Education, University of Palermo, 90128 Palermo, Italy; michele.roccella@unipa.it

**Keywords:** autism spectrum disorders, morphology, neurodevelopment

## Abstract

**Background**: Cranio-facial anomalies frequently occur in neurodevelopmental disorders because both face and brain are derived from neuroectoderm. The identification of differences in the facial phenotype of children with Autism Spectrum Disorders (ASD) may reflect alterations in embryologic brain development in children with ASD. **Methods**: we evaluated 33 caucasian children with ASD using a 2D computerized photogrammetry. Anthropometric euclidean measurements and landmarks located on the soft tissue of the face and head, were based on five cranio-facial indexes. Relationships between anthropometric z-scores and participant characteristics (i.e., age, Global IQ, severity of autistic symptoms measured using the CARS checklist) were assessed. **Results**: Cephalic index z-score differed significantly from 0 in our ASD group (*p* = 0.019). Moreover, a significant negative correlation was found between Facial Index z-score and CARS score (*p* = 0.003); conversely, a positive correlation was found between Interchantal Index z-score and CARS score (*p* = 0.028). **Conclusion**: our measurements shows a dolichocephalic head shape which is not correlated with autism severity. Importantly, two craniofacial markers were significantly correlated with autism severity: increased orbital hyperthelorism and decrease of height of the facial midline. These data support previous findings of craniofacial anomalies in autism spectrum disorder suggesting an “ASD facial phenotype” that could be used to improve ASD diagnoses.

## 1. Introduction

The neurodevelopmental model of autism spectrum disorders (ASD), postulates that the etiological origins of the disease can be traced to events in the prenatal period. In support of this neurodevelopmental model, higher rates of dysmorphic features, such as minor physical anomalies (MPAs) have been suggested as risk factors for autism and, correspondingly, as sensitive physical indicators of embryonic development [1,2,3].

MPAs are neurodevelopmental markers which manifest as unusual morphological features of the face or physique. MPAs can manifest as early as the first or early second trimester, and have consistently been linked to a biological vulnerability, which continues into adolescence and young adulthood. [2,4,5]

Autism spectrum disorders (ASDs) result from alterations in the embryological brain, suggesting that atypical development of the face or physique in ASD children, may result in subtle physical differences compared to typically developing children. MPAs remain stable over time and can be studied efficiently from early childhood onwards [1,3].

MPAs reported in ASD have been observed across multiple areas of the body, including the head, hands, and feet [3,4,5].

Rodier et al. [1] studied the frequency of individual MPAs in ASD and found that posterior rotation of the ear was more common in children with autism compared to the general population, as well as compared to children with developmental delays. Mixed results have been found for anomalies such as a high-arched palate and hyper-hypotelorism [3,4,5,6].

Of particular note, reoccurrence of anomalies in the craniofacial region suggests there may be facial characteristics related to subpopulations of ASD. Previous studies [6,7] on ASD face shapes, have shown that facial morphology differed significantly, between ASD and typically developing boys, and identified subsets of ASD boys, with distinctive craniofacial morphology. Specifically, decreased height of facial midline and long mouth widths were prevalent amongst individuals with ASD, with comorbid intellectual disability and greater severity of ASD symptoms. This could suggest an “ASD facial phenotype” representing a new physical biomarker that could be used to improve ASD diagnosis.

The face and brain are inherently linked in utero; their tissues share common origins, and develop in close coordination as a result of their physical adjacency and reciprocal molecular signalling [8]. Anthropometric photographic measurements represent an objective and quantitative methodology to characterize craniofacial MPAs in children with ASD. Additionally, photogrammetry can be used to detect generalized dysmorphology, in children who are often clinically difficult to examine [4].

The aim of the present study was to investigate the topographical pattern of cranio-facials anomalies in ASD children, and to determine whether these anomalies would correlate with intensity of ASD symptoms and overall functioning. More generally, assessment of MPAs may assist in the diagnostic protocol for neurodevelopmental disorders.

## 2. Material and Methods

### 2.1. Subjects

A total of 33 pre-pubertal children with ASD (28 males), between 4 and 12 years of age, were recruited by a specialized multidisciplinary team at Tours University Hospital (Tours, France). Ethical approval was obtained from the Ethics Committee of the Tours University Hospital. The study was performed in accordance with basic principles of the Declaration of Helsinki. An extensive, multidisciplinary child neuro-psychiatric assessment was carried out for each subject. Assessment consisted of a preliminary developmental history, medical examination and neuropsychological assessment. All subjects met the ICD-10 criteria for pervasive developmental disorders (PDD) according to the World Health Organization’s (WHO) International Classification of Diseases, 10th edition ICD-10). The sample consisted of Caucasian children of European origin, as normative physical data have been well-defined in the Caucasian population [9,10].

ASD was diagnosed using standardized diagnostic tools (mean age at assessment: 7.6 years; standard deviation SD: 2.2). The sample of ASD children included those who met diagnostic criteria for; childhood autism (F84.0, *n* = 22), atypical autism; (F84.1, *n* = 9), Asperger’s Syndrome (F84.5, *n* = 2) and other PDD (F84.9, *n* = 1). For inclusion, children were also required to score above threshold on all three domains of the ADI-R [11]. One child classified as having PDD not otherwise specified on the ADI-R (below threshold on 1 domain by 1 point) was also included. The intensity of ASD symptomatology was determined using the Childhood Autism Rating Scale (CARS) [12]. The CARS evaluates the severity of autistic behaviors in 14 functional areas by assigning a score from 1 to 4. An overall score is calculated by adding scores from all functional areas. It enables the assessment of severity of autistic symptoms, and the stratification of patients into three levels: “severely autistic” (score between 37 and 60), “mildly to moderately autistic” (score between 30 and 36.5), and “absence of ASD” (score less than 30). The questionnaire can be carried out in around 20–30 min.

Intellectual functioning was evaluated using the Weschler Abbreviated Intelligence Scale-III, (WAIS III) [13], allowing a global Intellectual Quotient (IQ), a non-verbal IQ (performance IQ) and a verbal IQ to be obtained for all ASD children.

In the routine course of evaluation, all ASD children received a comprehensive physical examination, to exclude the presence of a clinically detectible genetic syndrome associated with gross dysmorphic features and autistic-like symptoms. The examination was conducted by a pediatric team, in the same hospital site, examiners were blind to group status. Others exclusion criteria included clinical diagnosis of a co-morbid psychiatric or medical condition such as epileptic seizures or history of head injury.

Written informed consent was obtained from the parents or guardians of each participant.

### 2.2. Measurements

Cranio-facial examination of the ASD group employed a mixed approach of computerized photogrammetry and classic anthroposcopy. The examination was conducted by the same author (GT). Photographs were obtained using a high-resolution digital camera. The Euclidean measurements, based on anthropometric landmarks and 2D photogrammetric examinations, were obtained using image analysis software (FlashCAD^®^, Digitarch srl, Rome, Italy) [4]. Standardized cranio-facial photographs were taken with the camera lens aligned with the subject’s Frankfort horizontal plane [14]. An internal measure of scale (adhesive paper sticker) was placed on the glabella landmark for the frontal view, and on the condylion of the mandible for the profile view.

Subjects were encouraged to adopt a standardized expression: relaxed, not smiling, gently closed lips, eyes wide open. Spectacles were removed. After ensuring the participant was comfortable and able to sit still, collection of the images began. Multiple pictures of each child were taken to ensure that the image used for analysis adequately captured all of the facial areas required for landmarking.

**Figure 1:**
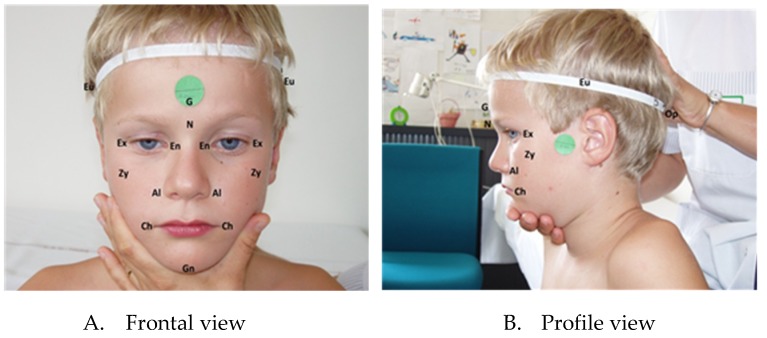
Standardized cranio-facial photographs with the camera lens aligned with the subject’s Frankfort horizontal plane and with an internal measure of scale (adhesive paper sticker) placed on the glabella landmark for the frontal view and on the condylion of the mandible for the profile view.

Anthropometric linear measurements and landmarks (Figure 1) on the soft tissue of the face and head, were based on five cranio-facial indexes, borrowed from the works of Farkas et al. [10] We selected cranio-facial indexes which required any Geodesic measurements (Table 1), due to previous findings that identification of facial phenotypes is independent from measurements type (Euclidean Vs Geodesic) [7].

Each skull-facial index was compared with the tables of anthropometric norms for Caucasian subjects, developed by Farkas et al. [10]. This enabled computation of *z*-scores. A synthesis of anthropometric characteristics of the sample is shown in Table 2.

## 3. Statistical Methods

Statistical analyses were carried out using Statistica V12 (TIBCO Statistica, Palo Alto, CA, USA). One-sample Student *t*-tests (T) were applied to compare anthropometric *z*-scores with 0. Relationships between anthropometric (i.e., Cephalic, Facial, Interchantal, Nasal and Mouse-face Index *z*-scores) and participant characteristics (i.e., age, sex, Global IQ, severity of autistic symptoms measured using the CARS) were assessed using Pearson’s product moment correlations and Student *t* tests. Where appropriate, standard multiple regression analyses were performed, to complement the interpretation of the relationships between each anthropometric variable (as dependent variable) and participant characteristics (as independent variables). Validity of regression analyses was tested according to multicollinearity, normal distribution and independence of residuals. In all cases, tests were performed on the two-sided 5% level of significance.

## 4. Results

**Figure 2:**
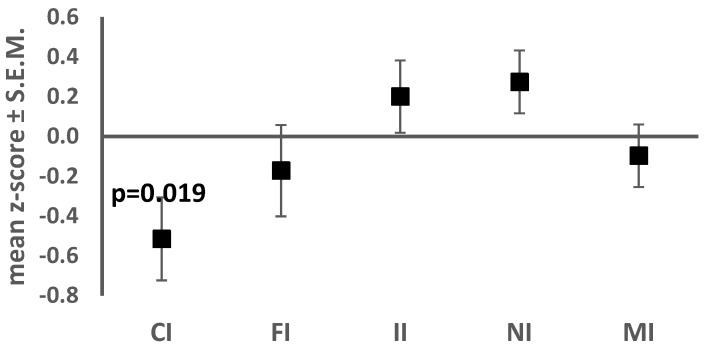
Anthropometric *z*-scores in the sample of 33 ASD patients (CI: Cephalic index, FI: Facial index, II: Interchantal index, NI: Nasal Index, MI: Mouth-face Index).

In a sample of 33 children with ASD, Cephalic Index was normal for 25 (75.8%) and subnormal (<−2SD) for 8 (24.2 %). The Facial Index was normal for 23 (69.7%), supernormal (>2SD) for 4 (12%) and subnormal for 6 (18.2%). The Interchantal Index was normal for 30 (91%) and supernormal for 3 (9%). The Nasal Index was normal for 30 (91%) and supernormal for 3 (9%). The Mouth-face Index was normal for 31 (94%) and subnormal for 2 (6%). Only Cephalic index z-score differed significantly from 0 (mean *z*-score: −0.51, 95% CI (−0.94, −0.09); *t* (32) = −2.46, *p* = 0.019). (Figure 2). Anthropometric *z*-scores are shown in Figure 2.

**Figure 3:**
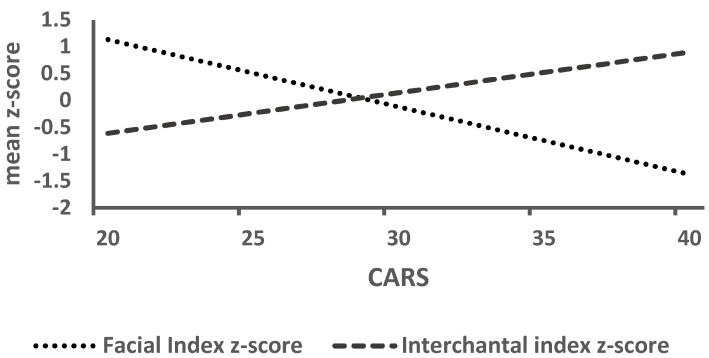
Estimated Facial and Interchantal index z scores related to Cars score.

A significant, large negative correlation was found between Facial Index *z*-score and CARS score (*p* = 0.003) (Table 3)—for every one unit increase in CARS score, the Facial Index *z*-score decreased by 0.1257 (Figure 3). Conversely, a positive medium correlation was found between Interchantal Index *z*-score and CARS score (*p* = 0.028) (Table 3)—for every one unit increase in CARS score, the Interchantal index increased by 0.075 (Figure 3). A medium correlation was also observed between Facial Index *z*-score and global IQ (*p* = 0.047) (Table 3). Moreover, no statistically significant difference was observed between boys and girls for the five index *z*-scores.

Since CARS score has been previously linked to intellectual functioning (Militerni 2002 et al. [15] in our study, *r* = −0.683, *p* < 0.001), standard multiple regression analyses were performed to clarify the relationships between Facial Index and Interchantal Index *z*-scores on one hand and CARS score and global IQ on the other (Table 4). Only the CARS score made a statistically significant contribution in explaining Facial Index *z*-score (*R*^2^ = 0.256, F(2,30) = 5.15, *p* = 0.012) and Interchantal Index z-score (*R*^2^ = 0.186, F(2,30) = 3.42, *p* = 0.046).

## 5. Discussion

In literature, craniofacial anomalies have been identified as putative biomarkers in providing insight into early neurodevelopment [8]. The causes of autism may be (epi)genetically determined, mildly associated with gene activity in early development or result from prenatal environmental risk factors. [16] Suggesting that a putative biomarker may well have neurodevelopmental origins. Furthermore, craniofacial anomalies are strongly associated with structural abnormalities in the brain, which are correlated with clinical severity in autism [17].

Our measurements, assessed by Euclidean distances between anatomical landmarks, show two craniofacial markers which are positively correlated with autism severity: increased orbital hyperthelorism and decrease of height of the facial midline. Conversely, our results on Cephalic index show a dolichocephalic head shape which does not correlate with autism severity.

Gray et al. [18] and Rajlakshmi et al. [19] pointed out that cephalic index is a gestational, age-dependent biometric parameter, in which intrinsic or extrinsic variations (unfavorable in pregnancy), may influence head shape between 14–28 weeks of gestation. New data from a retrospective study show atypical head growth trajectory during the in utero period, after the 22nd week of amenorrhea in children with ASD who showed postnatal head overgrowth. [20]. Moreover, a recent meta-analysis of studies of head size using head circumference, postmortem, and magnetic resonance imaging analysis, suggests a period of pathological enlargement restricted to the first 2–4 years of life, with rapid brain growth in the first 2 years and cessation of growth occurring between 2 and 4 years of age [21]. This suggests abnormal brain growth trajectory before brain enlargement. Head size measurements by orbital-occipitalis circumference was not a goal of our research. However, we can infer that dolichocephalic head shape in our ASD group may result from atypical antero-posterior cranial expansion, leading to larger head circumference in early childhood. Importantly, this finding is compatible with previous research, showing that brain and head size is a relatively non specific finding in autism [22] and that facial phenotypes were not driven by differences in head size [7].

Increased orbital hypertelorism is a common finding in our ASD group directly associated with autism severity.

In the literature, significantly increased inter-canthal and inter-orbital distances were found in ASD using the Waldrop Scale [4,23,24] or MRI [5] measurements. Referring to the early trajectory of optical development, Cheung et al. [5] shows that greater inter-orbital distance is linked with earlier head size expansion in autism. In their ASD group, they found that grey matter volume of the amygdala (extending bilaterally to its ventro-medial fusion to the head of hippocampus (unci) and inferior pole of superior temporal lobes) was positively correlated with inter-orbital distance.

Others studies have recently observed an enlargement of the amygdala in toddlers who are eventually diagnosed with autism spectrum disorders [25,26,27].

The link between increase in inter-orbital distance and increased amygdala volume, could be suggestive of early brain dysmaturation involving the amygdala. The amygdala is consistently implicated in socioemotional processing including; processing of emotions, emotion regulation, theory of mind, and eye gaze [28]. This suggests a link to “social brain” dysmorphology in autism spectrum disorders.

Our results show an inverse relationship between CARS score and Facial Index, supporting previous studies which found a link between increased severity of autism and reduced height of the facial midline [6,7]. These studies identified that decreased height of the facial midline and increase of mouth breadth were key biological traits in severe autism groups.

Contrary to our findings, the severe autism facial phenotype described by Aldrige et al. [7] includes a decrease in intercanthic distance. Previous studies have, similarly, described an association between smaller mean inter-orbital distance and intellectual disability in ASD groups [28].

Our findings support the concept that the processes governing the development of “social brain” structures (such as the amygdala) and optical differentiation from neural crest cells are likely to be tightly orchestrated. In the literature, decreased height of the facial midline is indicative of perturbations of midline structures of the human embryonic face [6]. As the embryonic notochord is a midline structure, midline cranio-facial development may also be compromised by aberrant brain development during fetal life. So increased inter-orbital distance in autism spectrum disorder can be explained by earlier antero-posterior trajectory expansion of the neurocranium.

Several limitations can be identified in the current study. The primary limitation of this study was a lack of some genetic data. ASD is a heterogeneous disorder, it does not have a single physical phenotype which would lead a physician to suspect an underlying genetic syndrome. Genetic testing methods, such as high-resolution karyotype and chromosomal microarray are now considered first-tier testing for individuals with ASD, developmental delay, and multiple congenital anomalies. However, these are not universally obtained by medical providers in clinical practice [29].

We included ASD patients without gross dysmorphic features, due to the direct association in literature between the severity of physical anomalies and increased likelihood of finding cytogenetic alterations [3]. On the other hand, new insights into craniofacial morphogenesis genes implicate them in the regulation of cerebro-craniofacial development [30]. Moreover, the multiple genetic and environmental influences on early life development, leading to clinical heterogeneity in ASD, could result in different cranio-facial (including optical) dysmorphology [5].

Our results have clinical relevance for medical ASD evaluation and treatment, as children with a distinctive craniofacial phenotype identifiable by 2D photogrammetry, may be considered candidates for further investigation of brain abnormalities and underlying genetic disorders.

The second limitation of this study was its relatively small sample size. Moreover, the study population was limited to Caucasian patients, as ethnicity can influence the prevalence of morphological abnormalities. Future studies are required to establish similar norms for other ethnic groups.

Thirdly, our methodology of 2D photogrammetry is limited to Euclidean measurement-although it has the advantage of being a significantly less expensive approach than 3D measurement techniques.

We assert that facial structure, based on two craniofacial markers-increased inter-orbital distance and decreased height of the facial midline - should be considered a potentially useful biomarker in predicting ASD severity. Using sophisticated facial phenotyping, based on two-dimensional photogrammetric imaging, our study found some distinctive facial characteristics related to ASD severity; these results corroborated previous studies, demonstrating the generalizability of facial phenotype as a viable biomarker for identifying ASD subgroups. Differences in facial morphology may reflect alterations in embryologic brain development in children with ASD.

## Figures and Tables

**Table 1:** Cranio-Facial Indexes.

Linear Measurements & Landmarks	Index Subnormal	Index Supernormal
Cephalic Index = eu-eu × 100/g-op	head narrow for its length	head wide for its length
Facial Index = n-ng × 100/zy-zy	face short for its width	face long for its width
Interchantal Index = en-en × 100/ex-ex	orbital hypotelorism	orbital hypertelorism
Nasal Index = al-al × 100/n-sn	nose narrow for its height	nose wide for its height
Facial-Mouth Width Index = ch-ch × 100/zy-zy	mouth narrow for face width	mouth wide for face width

**Table 2:** Demographic, clinical and anthropometric characteristics of the 33 children with ASD.

Demographic and Clinical Variables		Anthropometric Variables			
	Mean ± SD		Mean ± SD	*z*-score	Counts within Normal Range (%)
Age in months	91.5 ± 26.7	Cephalic Index	74.2 ± 5.1	−0.51 ± 1.20	25 (75.8)
CARS	30.4 ± 5.3	Facial Index	83.9 ± 6.6	−0.17 ± 1.32	23 (69.7)
Global IQ	59.9 ± 24.2	Interchantal index	38.7 ± 2.2	0.20 ± 1.04	30 (90.9)
Verbal IQ	50 ± 24.4	Nasal Index	72.5 ± 5.9	0.27 ± 0.91	30 (90.9)
Non-verbal IQ	70 ± 26.8	Mouth-face Index	36.7 ± 2.1	−0.09 ± 0.90	31 (93.9)

**Table 3:** Correlations between anthropometric *z*-scores and age at assessment, CARS, and Global IQ.

	Age		CARS		Global IQ	
	*r*	*p* Value	*r*	*p* Value	*R*	*p* Value
Cephalic Index *z*-score	0.330	0.061	0.316	0.073	−0.232	0.195
Facial Index *z*-score	−0.062	0.731	−0.506	0.003	0.349	0.047
Interchantal Index *z*-score	0.143	0.427	0.385	0.028	−0.122	0.499
Nasal Index *z*-score	−0.045	0.806	−0.275	0.122	0.138	0.445
Mouth-face Index *z*-score	0.001	0.997	−0.029	0.872	0.075	0.680

**Table 4:** Standard multiple regression analyses on facial Index and Interchantal Index *z*-scores, with CARS and Global IQ as dependent variables.

	Variable	Coef (SE)	*t* Value	*p* Value	95% CI
Facial Index *z*-score	CARS Global IQ	−0.125 (0.054) 0.000 (0.012)	−2.33 0.03	0.027 0.977	(−0.234, −0.015) (−0.024, 0.024)
Interchantal Index *z*-score	CARS Global IQ	0.111 (0.044) 0.011 (0.010)	2.51 1.17	0.018 0.250	(0.021, 0.566) (−0.008, 0.031)

Coef = unstandardized regression coefficient; SE = standard error; CI = confidence interval.
